# Flexible Fiberoptic Bronchoscopy: Indications, Diagnostic Yield and Complications

**DOI:** 10.7759/cureus.11122

**Published:** 2020-10-24

**Authors:** Sultan Qanash, Osamah A Hakami, Faisal Al-Husayni, Abdul Ghafoor Gari

**Affiliations:** 1 Internal Medicine, National Guard Hospital, King Abdulaziz Medical City, Jeddah, SAU; 2 Internal Medicine, Al-Imam Mohammed Ibn Saud Islamic University, Riyadh, SAU; 3 Internal Medicine, Umm Al-Qura University, Makkah, SAU

**Keywords:** flexible fiberoptic bronchoscopy (ffb), febrile neutropenia, hiv, mtb, chest infection, ild, hemoptysis, lung cancer, bal

## Abstract

Objective

Flexible fiberoptic bronchoscopy (FFB) has become an essential procedure for diagnosing and managing various respiratory conditions. We aimed to assess the main indications, diagnostic yield, and safety of FFB in our institute.

Methods

A total of 216 patients who underwent FFB between July 2009 and June 2012 at King Abdul-Aziz Medical City, Jeddah, Saudi Arabia, were reviewed retrospectively. Indications of the procedure, the diagnostic yield of variable respiratory diseases, and complications were reported.

Result

Out of 216 patients, 210 (97.2%) completed FFB. One hundred and ninety-eight (91.7%) bronchoscopies were for diagnostic purposes, and the remaining 12 (5.6%) were for therapeutic aim. The mean age of patients was 50 years ± 20 years and patients. Respiratory infection, malignancy, pulmonary infiltrate in febrile neutropenia (FN), and hemoptysis in order of frequency were the main indications. The overall diagnostic yield was 46%. Mycobacterium tuberculosis (MTB) was the most common indication (26.8%) and was identified in 37.7%, whereas pneumonia was confirmed in 46.3% of the patients. Malignancy was established in 35.3% of suspected cases, while eosinophilic pneumonia was diagnosed in 100%. The diagnostic yield in pulmonary infiltrates of human immunodeficiency viral (HIV) was 85.7%. Alveolar hemorrhage was the most common cause of hemoptysis. Out of therapeutic bronchoscopy, lung collapse was the main indication. There is no mortality and complications occurred in 1.5% of the cases.

Conclusion

MTB was the most common indication of FFB, followed by malignancy. It has a high diagnostic yield in eosinophilic pneumonia and pulmonary infiltrates in HIV patients. FFB is shown to be a safe modality for diagnostic and therapeutic purposes.

## Introduction

Flexible fiberoptic bronchoscopy (FFB) is an invasive procedure that has been used for a long time for diagnostic and therapeutic purposes. It contains a light source, fiber optics, and a camera that allows direct visualization of the upper and lower airways [1]. Generally, FFB is a safe procedure, which has a high diagnostic yield of respiratory disease. Its ability to assist in establishing the diagnosis has been improved with the presence of rapid technology revolutions. FFB plays a significant role in diagnosing various situations such as patients with hemoptysis, chest infection, parenchymal lung disease, lung nodules or masses, persistent lung infiltrates, mediastinal lymphadenopathy, and suspected lung transplant rejection [1,2]. Furthermore, FFB can be used therapeutically in cases of foreign body aspiration, ablation or debulking of endobronchial masses, airway stenosis, and lung lavage [2,3]. 

Multiple adverse effects may occur with FFB, which can be divided into medication- and procedure-related complications. Neuropathy, seizures, or even coma has been reported due to local anesthetics overdose [4]. Other medication-related complications include prolonged neuromuscular paralysis, hyperthermia, and hemodynamic instability [4,5]. On the other hand, procedural complications such as pneumothorax and bleeding can happen [5].

To enhance the outcomes of FFB procedures, an inter-professional team that excels in bronchoscopy is required. A skilled bronchoscopist, with the help of an endoscopy nurse and respiratory therapist trained in bronchoscopy equipment use, can decrease the complication rate [6]. The frequency of bronchoscopic indications is variable between previously conducted studies. Few studies have been done in the gulf area with varying numbers of patients and indications [2,7-9]. In this study, we aim to identify the indications and diagnostic yield of bronchoscopy procedures in diagnosing various respiratory diseases.

## Materials and methods

We reviewed a medical chart of 216 consecutive patients who underwent a bronchoscopic procedure between July 2009 and June 2012 at King Abdulaziz Medical City-Jeddah, Saudi Arabia. Written informed consent was obtained from each patient or patient's relative before the procedure. Data collected included demographic data, co-morbidity, bronchoscopic indications, and modality of bronchoscopy diagnosis, type and amount of sedation, radiological finding, bronchoscopic finding, and final diagnosis and bronchoscopic complications. A chest physician, thoracic surgeon, or fellow physician under consultant’s supervision performed bronchoscopic procedures. The bronchoscopic process was done in an equipped endoscopy unit; otherwise, unstable or intubated patients; the procedure was conducted in the intensive care unit (ICU). Vital signs were obtained before the procedure and monitored continuously during and after the procedure and all patients were observed post procedure until discharge. Supplemented oxygen was sufficiently given to all patients through a nasal cannula to maintain adequate oxygen saturation. Lidocaine with spray for pharynx and hypopharynx was given to all patients. An additional 2% of liquid lidocaine was instilled into the vocal cord and bronchial tree during the procedure. Other pre-medications, including midazolam, fentanyl, or pethidine, were given to most patients based on physician and patient’s status. All bronchoscopic procedures were done in a supine position with fiber-optic bronchoscopy (2.0-2.2-mm working channel; Olympus, Center Valley, PA, USA). Bronchoalveolar lavage (BAL) was obtained from all patients. Also, bronchial brushing, end-bronchial biopsy, or blind trans-bronchial biopsies were carried out according to clinical indication.

Statistical analysis

All data were entered on a computer using Excel version 2010. Descriptive statistics (frequency, mean, and stander deviation) of bronchoscopic diagnostic procedures were presented to describe the study variables. 

## Results

A total of 216 FFB procedures were completed within three years. Six procedures were aborted because patients were uncooperative (coughing, agitation, or not tolerating the procedure). Therapeutic interventions were done for 12 patients, and the remaining 198 procedures were performed for diagnostic aim. The mean age of patients was 50 years ± 20, with most males (57.4%). Table 1 shows the demographics of the participants.

**Table 1:** Patient demographics.

Age	50 years ± 20
Gender	N (%)
Male	124 (57.4%)
Female	92 (42.6%)
Chronic Diseases	N (%)
Diabetes	69 (31.9)
Hypertension	63 (29.2)
Heart Failure	19 (8.8)
Chronic kidney disease	22 (10.2)
Bronchial asthma	22 (10.2)
Chronic obstructive lung disease	15 (6.9)
Obstructive sleep apnoea	1 (.5)
Interstitial lung disease	2 (.9)

The mean dose of conscious sedation was: 2.03 mg ± 1.09 of midazolam, 48.4 ug ± 22.06 of fentanyl, and 27.78 mg ± 20.04 of pethidine. The mean duration of the FFB procedure was 20.7 minutes ± 10.17, while the mean recovery duration was 25.49 minutes ± 8.73. Diverse FFB modalities of diagnosis were completed: BAL in 100%, bronchial brush in 8.59%, and biopsy in 16.67%.

Table 2 demonstrates the indications of FFB. Diagnostic FFB accounted for 198 (91.7%) patients, while therapeutic FFB was done on only 12 (5.6%) patients, and six (2.8%) procedures were aborted. Respiratory infections were the most common indication of diagnostic bronchoscopy, with a total of 94 patients (47.5%) followed by lung cancer, with 34 (17.2%). Mycobacterium tuberculosis (MTB) was the most common cause out of chest infection, followed by pneumonia, which represented 26.8% and 20.7%, respectively.

**Table 2:** Indications of bronchoscopy MTB: mycobacterium tuberculosis; FN: febrile neutropenia; HIV: human immunodeficiency viral; ILD: interstitial lung disease.

Indications	
Diagnostic	n=198, N (%)
Respiratory infection	94 (47.5)
- Pneumonia	41 (20.7)
- MTB	53 (26.8)
Pulmonary infiltrate in FN	29 (14.6)
Pulmonary infiltrate in HIV	7 (3.5)
Suspected lung cancer	34 (17.2)
Suspected ILD	12 (6.1)
Hemoptysis	22 (11.1)
Therapeutic	n=12, N (%)
Lung collapse	6
Surgical intervention	5
Stridor	1
Aborted	n=6

The yields of diagnostic FFB are shown in Table 3. Overall the diagnostic yield of bronchoscopic procedures was 46%. On the other hand, the diagnostic yield of smear-negative MTB patients was (43.4%). It was confirmed by FFB procedure in 20 patients (37.7%), and the remaining three (5.7%) were diagnosed as chest infection other than MTB. Intervention modalities other than bronchoscopy (pleural tap, computed tomography-guided biopsy, and surgical intervention) were carried out in 11.3%, and MTB was confirmed only in four (66.8%) patients, whereas the other two patients were diagnosed with interstitial lung disease (ILD) and lymphoma. FFB procedure’s results were positive in 15 (44.1%) of suspected malignancy patients, and 20% of them were diagnosed with a chest infection. Six (17.7%) of suspected malignancy patients were confirmed as lung cancer by modality other than bronchoscopy and one (2.94%) with mycobacterium other than MTB. The overall diagnostic yield of pneumonia patients was 53.7%, but 7.3% of them were identified as MTB. Pulmonary infiltrates in febrile neutropenia (FN) patients represented 14.6% of diagnostic FFB, and the positive yield was in four (13.8%) patients only. All of them were confirmed as a chest infection. Two out of four were diagnosed as Cytomegalovirus (CMV) pneumonitis and another two as fungal pneumonia. Also, pulmonary infections were confirmed in the majority of HIV patients (85.7%). *Pneumocystis jirovecii* pneumonia (PCP), CMV pneumonia, and fungal pneumonia were the primary diagnosis in those patients and represented 66.7%, 16.7%, and 16.7%, respectively. Approximately 6% of diagnostic procedures were done for suspected ILD, and the diagnosis was confirmed in 58.3% of them. Eosinophilic pneumonia was suspected in five (41.7%) ILD patients, and the diagnosis was established in all of them by bronchoscopy. The diagnosis of hemoptysis was reached in 13 patients (63.6%). Alveolar hemorrhage in five (38.5%) and malignancy (30.8%) were the principal diagnoses.

**Table 3:** Diagnostic yield by indication, MTB: mycobacterium tuberculosis; FN: febrile neutropenia; HIV: human immunodeficiency viral; ILD: interstitial lung disease.

Indications	Total no.= 198 N (%)	Diagnostic yield n=91 N (%)
Pulmonary infiltrate in FN	29 (14.6%)	4 (13.8%)
Pulmonary infiltrate in HIV	7 (3.5%)	6 (85.7%)
Suspect ILD	12 (6.1%)	7 (58.3%)
Suspect Malignancy	34 (17.2%)	15 (44.1%)
MTB	53 (26.8%)	23 (43.4%)
Respiratory infection	41 (20.7%)	22 (53.7%)
Hemoptysis	22 (11.1%)	14 (63.6%)

The diversity of radiological appearances in our patients were detected, as demonstrated in Table 4. Air space disease pattern was the most common radiological finding in chest x-ray (51.5%) and high-resolution computed tomography (HRCT) (44.4%). Complications took place in three patients who underwent diagnostic bronchoscopy (1.5%). Hypoxia was observed in wo (1%) patients, and one patient (.5%) had bleeding.

**Table 4:** Radiological appearance of patient who underwent diagnostic bronchoscopy. HRCT: high resolution computed tomography.

Findings	Chest X-ray N (%)	HRCT N (%)
Normal	14 (7.1%)	3 (1.5%)
Air space disease	102 (51.5%)	88 (44.4%)
Interstitial pattern	22(11.1%)	33 (16.7%)
Cavity	3 (1.5%)	9 (4.5%)
Mass	11 (5.6%)	18 (9.1%)
Mediastinal lymph node	9 (4.5%)	9 (4.5%)
Other	37 (18.7%)	38 (19.2%)

## Discussion

FFB is an assisting tool in the diagnosis and management of a variety of respiratory diseases. Our study found that the most common indication was to diagnose respiratory infections, mainly MTB. The overall diagnostic yield was 46%, with the highest yield in HIV patients with pulmonary infiltrates. The complication rate was low, as it only occurred in three patients. Given the difference in studies sitting and populations, the bronchoscopy procedure's overall diagnostic yield is variable between studies. The range of yield, reported from previous studies, was between 44% and 65% [8,10]. The overall diagnostic yield of our research is almost similar to what has been reported.

Although the indications of bronchoscopy have been the same since the emergence of bronchoscopy, these indications' frequency was variable among the studies. In Saudi Arabia, two studies were conducted by Alamoudi et al. [7] and Alzeer et al. [8] showed that chest infection was the most common indication with 31% and 35.9%, respectively, followed by malignancy with 19% and 25.9%, respectively. In addition, chest infection included MTB was reported as a major indication in neighboring countries [9]. In our study, the two most common indications agreed with these studies as chest infection represented almost half of the cases while lung cancer came second with 17.2%. However, Taha et al. reported that malignancy was the most common indication in his study [10]. Western studies have conflicting results as some categorized malignancy as the most frequent indication [11,12], while others reported that pneumonia is the leading indication to proceed with bronchoscopy [13].

MTB's incidence rate has a rising manner in our area, despite significant practice to control this disease [14]. The annual incidence rate of MTB in Saudi Arabia ranges between 14 and 17/100,000, with the highest rates in Makkah region where our study took place [15]. Moreover, multi-resistant MTB accounts for 4.4% while mono-resistant has a 3.8% rate, emphasizing the importance of rapid detection of the organism [16]. Early diagnosis of pulmonary MTB can help disease prevention and progression as well. However, bronchoscopy has an integral part in the diagnosis of negative smear MTB patients. Previous studies revealed a wide range of bronchoscopy diagnostic yield in negative smear TB patients, which was reported as low as 10% [17-18]. In our study, MTB diagnosis was confirmed in 37.7%, higher than previous studies, yet lower than reported by Alzeer et al. (67%) [8]. As Saudi Arabia is considered an endemic area, physicians tend to have a high index of MTB suspicion in patients presenting with respiratory symptoms, and consequently, this factor led to a lower threshold to proceed with bronchoscopy procedure.

The diagnosis of pulmonary infiltrates in FN patients is difficult and challenging. Bronchoscopy is a standard tool to reach a diagnosis in those patients. Nonetheless, various studies have been done to determine the efficacy of the bronchoscopic procedure in this setting. The reported diagnostic yield was ranging between 15-67% [19-21], which is higher, to some extent, than our diagnostic yield. We attribute lower diagnostic yield in our patients to two reasons. All of our FN patients were on antimicrobial agents, and some of them were on antifungal medications, and there were no other than BAL modalities performed on those patients. 

Respiratory disease has a significant impact on HIV patients, and bronchoscopic procedure creates excellent value in diagnosing chest infiltrates. It has high sensitivity in diagnosing PCP in HIV patients [22-24]. Our data showed an overall high diagnostic yield of bronchoscopic procedures in those patients. Interestingly, our findings suggest that bronchoscopy has a relatively high diagnostic yield in suspected cases of ILD, with eosinophilic pneumonia representing the majority. Diagnosis of eosinophilic pneumonia is made by the presence of alveolar eosinophilia ≥ 25% in BAL with correlated clinical and radiological findings [25]. Striking eosinophilia counts in BAL differentiate between eosinophilic pneumonia and other ILD [26]; accordingly, we used these criteria to define our eosinophilic pneumonia patients.

Hemoptysis is one of the leading and alerting respiratory symptoms, and it should be considered a life-threatening condition that warrants effective treatment and assessment [27]. The bronchoscopic procedure may differentiate the possible causes, such as end bronchial tumors, chest infection, and alveolar hemorrhage [28]. Moreover, it has a role in identifying the source of bleeding [29]. Alveolar hemorrhage has a distinctive feature during bronchoscopy procedure with more bloody sequential aliquot, accompanying the presence of hemosiderin-laden macrophage in histopathology of BAL as well [30]. This distinctive feature was identified in five patients in our study.

Similar to any medical field procedure, the bronchoscopic method has minor and major complications [7,12]. Notwithstanding, it is a safe procedure, even with its capability to get mediastinal lymph node and lung biopsy. This practice's safety was documented in our study, as only 1.5% of our patients had complications due to this procedure.

## Conclusions

Chest infections, mainly smear-negative MTB, are the most common indication of bronchoscopy procedures, despite high investment in disease prevention. FFB has a crucial importance rule in diagnosing this illness and other respiratory diseases. Moreover, it’s a safe procedure for various patients with respiratory diseases with high diagnostic yield. 
